# Relationship between CAINS negative symptoms and cognition, psychosocial functioning and quality of life in patients with a first psychotic episode of schizophrenia

**DOI:** 10.1192/j.eurpsy.2023.1334

**Published:** 2023-07-19

**Authors:** R. Rodriguez-Jimenez, L. García-Fernández, V. Romero-Ferreiro, M. Valtueña García, A. I. Aparicio, J. M. Espejo-Saavedra, L. Sánchez-Pastor, A. Nuñez-Doyle, M. Dompablo, O. Jiménez-Rodríguez, D. Rentero, P. Fernández-Sotos, I. Martínez-Gras, J. L. Santos

**Affiliations:** 1 Psychiatry, Hospital Universitario 12 de Octubre; 2 Psychiatry, Universidad Complutense de Madrid; 3 CIBERSAM, Madrid; 4 Psychiatry, Univesidad Miguel Hernandez, Alicante; 5 Quality and Academic Compliance Unit, Universidad Europea de Madrid, Madrid; 6 Psychiatry, Hospital Virgen de la Luz, Cuenca; 7 Salud Mental, Complejo Hospitalario Universitario de Albacete, Albacete, Spain

## Abstract

**Introduction:**

Negative symptoms has been classically associated with cognition, psychosocial functioning and quality of life in patients with schizophrenia. But negative symptoms are not a unitary construct, encompassing two different factors: diminished expression, and motivation and pleasure. Few works have studied the relationship between these two different negative symptoms factors and cognition (neuro and social cognition), psychosocial functioning and quality of life, jointly, in patients with a first psychotic episode of schizophrenia.

**Objectives:**

The objective of the present work was to study, in a sample of patients with a first psychotic episode of schizophrenia, the relationship between the negative symptoms (diminished expression and motivation and pleasure) and neurocognition, social cognition, functioning and quality of life.

**Methods:**

The study was carried out with 82 outpatients with a first psychotic episode of schizophrenia from two Spanish hospitals (“12 de Octubre” University Hospital, Madrid and “Virgen de la Luz” Hospital, Cuenca). The patients were assessed with the Clinical Assessment Interview for Negative Symptoms (CAINS) for evaluating diminished expression (EXP) and motivation and pleasure (MAP) symptoms, the MATRICS Consensus Cognitive Battery (MCCB) for evaluating neurocognition and social cognition, the Social and Occupational Functioning Assessment Scale (SOFAS), and the Quality of Life Scale (QLS).

**Results:**

A negative correlation was found between neurocognition and the two negative symptoms subscales: CAINS-EXP (r=-0.458, p<0.001) and CAINS-MAP (r=-0.374, p<0.001); but with social cognition only CAINS-EXP was correlated (r=-0.236, p=0.033). Also, it was found a high negative correlation between SOFAS scores and CAINS-MAP (r=-0.717, p<0.001); and a medium negative correlation with CAINS-EXP (r=-0.394, p<0.001). Finally, QLS score was high correlated with both CAINS subscales: CAINS-EXP (r=-0.681, p<0.001) and CAINS-MAP (r=-0.770, p<0.001).

**Conclusions:**

This study found a relationship between negative symptoms and neurocognition, social cognition, functioning and quality of life in a sample of patients with a first psychotic episode of schizophrenia. But the two different negative symptom factors, diminished expression, and motivation and pleasure, are associated differently with psychosocial functioning, but especially with social cognition where the relationship was only found with diminished expression symptoms.

**Disclosure of Interest:**

None Declared

